# Epstein–Barr Virus Reactivation-Induced Immunoglobulin Production: Significance on Autoimmunity

**DOI:** 10.3390/microorganisms8121875

**Published:** 2020-11-27

**Authors:** Keiko Nagata, Kazuhiko Hayashi

**Affiliations:** 1 Division of Pharmacology Faculty of Medicine, Tottori University, 86 Nishi-Cho, Yonago 683-8503, Japan; 2 Department of Pathology, Faculty of Medicine, Tottori University, 86 Nishi-Cho, Yonago 683-8503, Japan; hayashik@tottori-u.ac.jp

**Keywords:** Epstein-Barr virus (EBV), reactivation, autoimmune disease, Graves’ disease, thyrotropin receptor antibody (TRAb), IgM, complement

## Abstract

Epstein–Barr virus (EBV) mainly persists in B cells, which differentiate into antibody-producing cells, and thus, EBV has been implicated in autoimmune diseases. We aimed to describe the EBV reactivation and its relevance to autoimmune disease, focusing on Graves’ disease, which is an autoimmune hyperthyroidism caused by thyrotropin receptor antibodies. Circulating autoreactive B cells that have evaded from the selection have difficulties differentiating to produce antibodies. However, once EBV infects such B cells and reactivates, the B cells may become plasma cells and produce autoantibody. We herein proposed an EBV reactivation-induced Ig production system, which is a distinct pathway from the antibody production system through germinal centers and bone marrow and has the following characteristics: 1. IgM dominance, 2. ubiquitous Ig production, and 3. the rescue of autoreactive B cells, which skews Ig production toward autoantigens. IgM autoantibodies induced by EBV reactivation may activate the classical complement pathway and injure healthy tissue, which supply autoantigens for the production of affinity-matured IgG autoantibodies. Antibodies induced by EBV reactivation may play important roles in the development and exacerbation of autoimmune diseases.

## 1. Introduction

Epstein–Barr virus (EBV) has been implicated in autoimmune diseases [1,2,3,4]. Epstein–Barr nuclear antigen (EBNA)1 was previously shown to exhibit cross-reactivity with the myelin antigen of multiple sclerosis (MS) [5] or with the Ro (ss-A) protein, which appears prior to the onset of systemic lupus erythematosus (SLE) [6], and patients with a high EBNA1 titer were found to be at risk of developing MS and SLE. Despite these epidemiologic evidences, a pathophysiological link between EBV and autoimmune disease remains controversial.

Recently, the effectiveness of B cell depletion therapy by anti-CD20 monoclonal antibodies had been shown on rheumatoid arthritis (RA), SLE and MS [7,8,9]. Thus, importance of the B cell-tropic EBV on the pathophysiology of autoimmune diseases has become highlighted. In addition, one of the common features of autoimmune disease is tissue injury [9,10,11].

Infectious mononucleosis (IM) is a symptomatic primary infection of EBV. Acute EBV infection is known to induce polyclonal B cell activation [1,12,13]. Various autoantibodies have been detected in the acute phase serum of IM patients and autoimmune disorders may develop after IM [14,15,16,17]. We also noted some reports that had detected autoantibodies released from lymphocytes infected with exogenously-added EBV [18,19,20,21]. Tamoto et al. recently reported that serum thyrotropin receptor antibodies (TRAbs) may be present even in asymptomatic EBV primary infection [22]. These findings support a relationship between EBV infection and autoimmunity.

## 2. EBV and Its Reactivation

EBV is a common human herpes virus [1] that was discovered by Epstein et al. in 1964 in a culture of Burkitt lymphoma cells. It is also known as the first human oncovirus. Its genome is double-stranded DNA of approximately 170 kbp.

In primary infection, EBV infects B cells directly or through oropharyngeal epithelium [1,23]. Most of the infected B cells become latently infected, and some of them become lytic and produce infectious virions [1,23]. Some latently infected B cells return to the oropharynx, become lytic, and release the virus. After primary infection, EBV mainly persists in B lymphocytes for life.

The characteristic proteins expressed in EBV latency are the EBNAs and latent membrane proteins (LMPs) (Table 1). In addition to the proteins, EBV-encoded small RNAs (EBERs) and microRNAs are expressed. EBNA1 is necessary to retain and replicate the genome of EBV through its action as a bridge coupling the EBV genome to host chromosomes [24]. EBNA2 plays essential roles in the transformation of infected cells [25]. It has also been shown to up-regulate the promoter of LMP1 [26], which is critical for B-cell activation. LMP1 is known to mimic host CD40 signal constitutively [12]. Therefore, the cell activation pathway including nuclear factor κB (NF-κB) is promoted, and further, LMP1 induces the expression of bcl-2, which supports the cell survival and growth. On the other hand, LMP2A constitutively mimics signaling from the B-cell receptor (BCR) [27].

EBV-encoded small RNAs (EBERs) are more abundant than any other EBV-related antigen, of which there are 10^7^ copies per cell [1]. Furthermore, EBER1 is shown to be approximately 10-fold more abundant than EBER2.

In latently infected cells, the EBV genome is replicated once per cell cycle by host DNA polymerase. However, occasionally, EBV may be lytically reactivated, and its lytic genes are sequentially expressed, and lytic replication cycles are induced (Table 1). BCR stimulation is known to induce lytic replication from latency [28]. The plasma differentiation of the host B cell is also related to the lytic reactivation [29,30]. BZLF1 and BRLF1 are the transactivators expressed in the immediate early period of lytic replication [1] (Table 1). They activate promoters of early lytic genes. Early lytic genes encode proteins related to viral replication. The protein encoded by BMRF1 is called early antigen (EA), which functions as a polymerase-associated processivity factor [1]. BALF5 and BGLF4 encode DNA polymerase and virion protein kinase, respectively [1]. The product of late lytic genes includes structural protein. The expression of these lytic genes occurs sequentially, and a large number of infectious virions are released, which deteriorate host cells [1,24,28]. Grimm-Geris et al. reported that 45.1% of healthy university students shedded EBV in their gingival swab [31], which showed the lytic replication occurred in a healthy, non-immunosuppressed state.

## 3. Graves’ Disease Is an Autoimmune Thyroid Disease

In our study of EBV reactivation and autoimmunity, we adopted Graves’ disease, an autoimmune hyperthyroidism, as an example, because the number of patients of Graves’ disease is large, and consequently many efficient antibodies or receptor proteins were available. The mechanisms we demonstrate here may be common in every autoimmune disease, and therefore, we would like to explain about the EBV-reactivation-induced Ig production and autoimmunity with Graves’ disease.

Graves’ disease accounts for the majority of hyperthyroidism cases [32]. Patients with Graves’ disease have a diffusely enlarged thyroid gland and elevated serum thyroid hormone levels. They develop palpitations, dyspnea, heat intolerance, or weight loss because thyroid hormone activates the sympathetic nervous system, calorigenesis, and metabolism.

**Figure 1:**
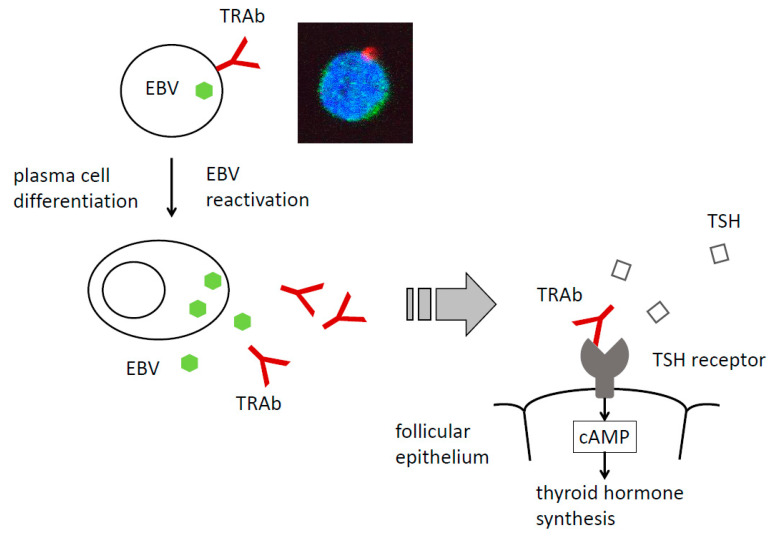
EBV reactivation induces plasma cell differentiation and the production of thyrotropin receptor antibodies (TRAbs). The reactivation of EBV persisting in TRAb-producing cells induces plasma cell differentiation and TRAb production. TRAb bind the thyrotropin receptor (TSHR) competitively with thyrotropin (TSH) and stimulates the thyroid follicular epithelium. Inset: A TRAb (red) and EBER1 (green) double-positive cell.

Thyrotropin receptors (TSHRs) on the surface of the thyroid follicular epithelium bind thyrotropin (thyroid-stimulating hormone: TSH) secreted from the pituitary gland and signals including cAMP for thyroid hormone production are transmitted [33,34] (Figure 1). Patients with Graves’ disease have TSHR autoantibodies called TRAbs that bind TSHR competitively with TSH. Although TRAbs are heterogeneous antibodies, most are the stimulating type [32,35,36]. Weetman et al. reported that stimulating TRAbs are restricted to an IgG1 subclass, since only IgG1 fractions increased cAMP [32,37]. In their experiments, non-IgG fractions consisted mainly of IgM, but they never increase cAMP. On the other hand, Kraiem et al. reported that TSHR-blocking antibodies were distributed in various IgG subtypes and suggested that they were polyclonal [38]. These results are not conflicting with the report of Furmaniak et al. showing that TRAb-IgG contained both stimulating and blocking antibodies [39]. Other groups established several TRAb-producing B cell clones from patients of Graves’ disease with EBV, of which most turned out to be in IgM classes. The explanation of these results may be that their samples were EBV-transformed lymphoblastoid cell lines (LCLs).

Thyroid diseases with lymphocytic infiltration of the thyroid gland and characteristic autoantibodies against thyroid components are called autoimmune thyroid diseases (AITD) [32,40,41]. Graves’ disease and Hashimoto’s thyroiditis are representative AITD, and TRAb is the autoantibody for Graves’ disease, while the anti-thyroid peroxidase (TPO) antibody and anti-thyroglobulin antibody are autoantibodies for Hashimoto’s thyroiditis. An insult that leads to an immune response may be important as the mechanism for AITD.

Graves’ disease and Hashimoto’s thyroiditis may simultaneously occur in the same patient and within the same family. Patients with Graves’ disease are at an increased risk of other autoimmune diseases, including insulin-dependent diabetes mellitus, myasthenia gravis, Sjögren’s syndrome, and RA [32]. Genetic factors and environmental factors have been proposed as risk factors for Graves’ disease. An iodine intake, stress, or infection may be environmental factors. However, an emphasis has been placed on genetic (hereditary) factors based on previous findings showing that siblings had a high risk of being affected and monozygotic twins had a higher concordance rate than dizygotic twins [32,41,42].

Although many susceptible genes have been reported to date, their risk ratios were not high: risk ratio of twofold to fourfold [32]. We herein proposed viral infections as an important environmental factor, namely, Epstein–Barr virus (EBV) infection.

## 4. EBV Lytic Reactivation Stimulates Antibody Production by Host B Cells

A product of the *BMRF1* gene called EA is expressed in the early replicative cycles of lytic reactivation (Table 1). It has been used as the marker of EBV reactivation for the convenience of measurement and sample storage. However, EBV reactivation could occur in healthy subjects [31]. Therefore, we must be careful in explaining the results of EA antibody levels.

We previously reported a correlation between serum TRAb levels and EA antibody levels in 66 patients with Graves’ disease [43]. As healthy controls in this study, we used healthy laboratory staff other than hospital staffs who may have night duty.

B cells differentiate into antibody-producing cells (plasma cells). When EBV persists in autoreactive B cells, EBV latency or reactivation may influence antibody production. Previous studies suggested a relationship between plasma cell differentiation and EBV reactivation [29,30]. We hypothesized that the reactivation of EBV persisting in TRAb-positive B cells may stimulate the plasma-cell differentiation and production of TRAbs, thereby inducing or aggravating Graves’ disease (Figure 1).

### 4.1. Detection of EBV-Infected B Cells That Have Autoantibodies on Their Surface

Pathologists detect EBV-infected cells on tissue slides using an EBER1 peptide nucleic acid (PNA) probe labeled with fluorescein isocyanate (FITC), and this technique is called EBER1 in situ hybridization (ISH) [44]. Fundamentally, EBERs were found to be expressed in latently infected cells; however, the in vitro induction of lytic reactivation did not reduce the expression of EBERs [45]. Kimura et al. developed the application of EBER1 ISH for floating cells [46].

We sampled PBMCs from eight patients with Graves’ disease and eight normal controls, and stained surface TRAb by recombinant human TSHR and anti-human TSHR antibody to the C terminus. Then, we stained EBER1 using the methods described above. Flow cytometry revealed the presence of TRAbs and EBER1 double-positive cells (TRAb(+) EBV(+) cells) in all patients and control samples (Figure 1) [47].

### 4.2. Autoantibody Production Induced by EBV Reactivation

We then induced the reactivation of EBV on PBMCs containing TRAb(+) EBV(+) cells. Various strategies have been shown to induce EBV lytic reactivation, including phorbol esters and calcium ionophores. We used a culture at 33 °C to induce reactivation, which was moderate, but physiological because no chemicals were used [48,49,50].

During EBV reactivation, we detected TRAbs released in the culture fluids of each sample [51]. Reactivated cells contained CD138(+) cells, which exhibited compatible characteristics with plasma cells. These cells had the glycoprotein gp350/220, an EBV reactivation late gene product (Table 1), on their surface. Released TRAbs bound to TSHR and its levels were measured using a radio-receptor assay.

Studies showing EBV-infected B cells mimicing the pathway in which naive B cells differentiate to become resting memory B cells are controversial [1], and Thorley–Lawson et al. have stated that EBV-infected B cells enter germinal centers and differentiate to resting memory B cells [52]. However, in our results, the B cells differentiated to be plasma cells and secreted antibodies without germinal centers.

These findings primarily suggest that TRAb(+) EBV(+) cells are present in peripheral blood, and EBV reactivation induces TRAb secretion from these cells.

## 5. Difference between Patients and Controls

Our findings showing that not only patients with Graves’ disease, but also healthy controls, have TRAb(+) EBV(+) cells, which indicates that both have autoreactive TRAb(+) cells that evaded central selection in bone marrow and may have partially escaped peripheral selection. However, these cells in healthy controls did not produce TRAbs in vivo to sufficient levels for a clinical cut-off; however, patients with Graves’ disease tested positive for serum TRAbs. This difference between patients and controls appeared to depend on the persisting strain of EBV, as well as on the genetic factor. EBV persisting in the B cells of patients may have been more easily reactivated than that in healthy controls.

EBV is divided into two major strains: type A (1) and type B (2), with type A being more dominant worldwide [1]. The genes and amino acid sequences of EBNA-LP, EBNA2, EBNA3A, EBNA3B, EBNA3C, and gp350/220 (Table 1), and the numbers of various repeats have been shown to markedly differ between the two strain types [1,53,54]. EBNA2 plays essential roles in the transformation of infected cells [25,55]. It has also been shown to up-regulate the promoter of latent membrane protein (LMP) 1 [26], which is critical for B-cell activation. Though the ability to transform in vitro could not explain all of the mechanisms of autoimmunity, the efficiency to establish infection appears to depend on the EBV strain. Many infectious virions are released during the induction of reactivation. EBNA2 and LMP1 are necessary for virions to establish infection in surrounding cells and expand the infected cell population.

Whole-genome sequencing showed that the lytic genes for reactivation (Table 1) were conserved between type A and type B [56]. However, the splicing variant of BZLF1, an immediate-early gene product, was recently reported in EBV(+) epithelial cell lines [57]. Therefore, some mutations in lytic genes may be associated with reactivation.

Regarding the frequency of TRAb(+) B cells, by the time B cells enter the circulation, they may be defined genetically; the strain of persistent EBV is also important for the expansion of the TRAb(+) EBV(+) cell population and TRAb production.

## 6. Mechanisms of EBV Reactivation-Induced Ig Production

### 6.1. EBV Reactivation Also Induces Class-Switched Antibodies

**Figure 2:**
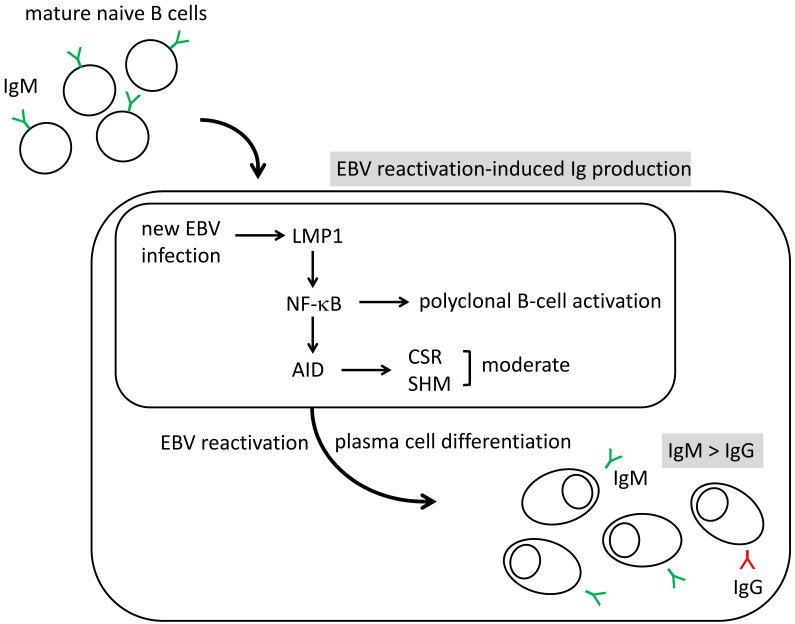
IgM dominant production induced by EBV reactivation. In EBV reactivation-induced Ig production, newly infected EBV induces LMP1 and then stimulates NF-κB to activate polyclonal B cells. NF-κB binds to the promoter of the AID gene (AICDA) to stimulate AID production. Class-switch recombination (CSR) and somatic hypermutation (SHM) may be catalyzed, but are moderate. Therefore, the Ig produced are IgM-dominant.

We induced EBV reactivation in PBMCs from Graves’ disease patients and healthy controls cultured at 33 °C according to our previous method [48,49,50]. We detected various isotypes of immunoglobulins (Igs): IgG, IgM, and IgE [58]. We also identified the expression of activation-induced cytidine deaminase (AID), which catalyzes class-switch recombination (CSR) and somatic hypermutation (SHM) [59] (Figure 2), and noted that the expression of *AICDA* (the AID gene) increased on day 5 of the induction of reactivation. Immunohistochemistry on culture cells on day 12 confirmed the strong staining of the AID protein.

Heath et al. had reported that AID transcripts upregulated in EBV-transformed LCL [60]. They had detected SHM by the sequencing of immunoglobulin heavy chain variable region (IgHV) genes, but they observed that their LCL from naive B cell or non-switched memory B cells had not undergone CSR, because the LCLs presented IgM+ IgD+ phenotype.

The rates of peripheral B cells with surface globulin other than IgM are approximately 15% [61]. In our study, at day 12 of EBV reactivation induction, the rate of released IgG and IgE in total was approximately 40%. Therefore, we considered the CSR as functioning, even if it was mild

Heath et al. also stated that the LCL changed the Ig class to IgG by adding CD40L and IL-4 stimulation [60]. In our study, the T cell functions were suppressed by cyclosporine A, but at the beginning, T cells were present and could produce IL-4. Furthermore, EBV-LMP1 mimics the CD40 signal of the host B cells, therefore, CSR could function in our culture.

**Figure 3:**
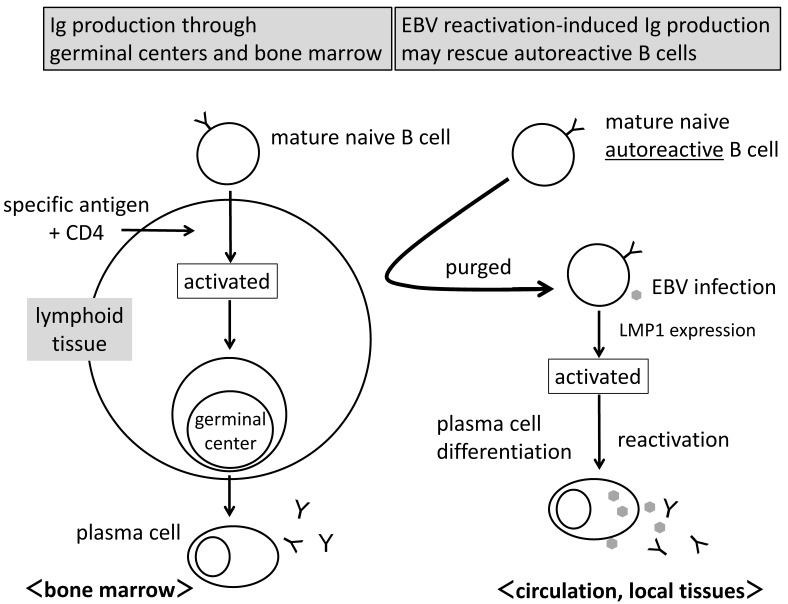
Rescue of autoreactive B cells. Left: Pathway of Ig production through germinal centers and bone marrow. B cells are activated by the presentation of their specific antigens for cognate CD4 T cells and a CD40 signal. Activated and proliferated B cells form germinal centers to affinity-matured class-switched antibodies in bone marrow. Right: Autoreactive B cells that have difficulty entering lymphoid tissue may activate and produce antibodies through EBV reactivation-induced Ig production.

The binding of the transcription factor NF-κB to the *AICDA* promoter is important for the expression of AID [13,62]. B cells typically encounter their specific antigen and are activated by receiving the CD40 signal from cognate CD4 T cells following the presentation of the digested antigen to T cells, and the CD40 signal then activates NF-κB (Figure 3, left).

EBV-LMP1 is known to constitutively mimic the CD40 signal [12]. Therefore, EBV plays a role in the CD40 signal by inducing LMP1 and activating NF-κB without a specific antigen and cognate CD4 T cells. Activated NF-κB then initiates the transcription of *AICDA*.

### 6.2. EBV Infection Causes Polyclonal B Cell Activation

In addition to NF-κB, LMP1 induces the cell activation pathway; MAPK, JNK, PI3K/Akt, and IRF7 [1]. Therefore, EBV activates host B cells through the expression of LMP1. B cells may be non-specifically activated by random infection and the spreading of EBV. Consequently, polyclonal B cell activation may be induced in acute EBV infection.

During EBV primary infection, the majority of infected cells become latency type 3 and express LMP1 on the plasma membrane [23] (Figure 2). In EBV reactivation, the original host B cell will deteriorate; however, many new virions are produced and enter the surrounding cells. These newly infected cells also become latency type 3 and express LMP1. Therefore, in EBV reactivation and new infection, LMP1 induced on the plasma membrane mimics the CD40 signal and activates NF-κB, and infected B cells are then polyclonally activated [1,12,13]. NF-κB stimulates B cells to express AID. The continuous stimulation of EBV reactivation induces the differentiation of B cells to plasma cells and antibody production. Therefore, we proposed “EBV reactivation-induced Ig production system” (Figure 2).

### 6.3. EBV Reactivation-Induced Ig Production as an Alternative System

B cells enter the circulation after antigen specificity has been established in bone marrow (mature naive B cells) [63]. B cells activated by the presentation of their specific antigen to cognate CD4 T cells proliferate and migrate into primary follicles to form germinal centers (Figure 3, left). Mature naive B cells with IgM undergo CSR and SHM, with the majority producing affinity-matured IgG. Only cells that survive this selection process become memory B cells or plasma cells. Plasma cells then migrate to bone marrow to secrete high-affinity isotype-switched antibodies for a long period of time [64].

In contrast to the antibody-producing system through germinal centers and bone marrow, when EBV infects circulating mature naive B cells, these B cells may be activated polyclonally and express AID by signals from LMP1 (Figure 3, right). This activation may occur without specific antigens and the assistance of CD4 T cells. EBV reactivation then induces the terminal differentiation to plasma cells and antibody production. EBV reactivation-induced Ig production may be an alternative antibody-producing system.

### 6.4. Characteristics of EBV Reactivation Induced Ig Production

#### 6.4.1. IgM Dominance

The majority of antibodies released in response to the induction of EBV reactivation in a culture were found to be IgM [58]. AID induced by LMP1 may function moderately. EBV may infect both memory and mature naive B cells [1], and 70–90% of circulating B cells are mature naive B cells with IgM [19,20,61,63]. Since EBV randomly infects circulating B cells, the antibody produced may be IgM dominant, even if the induced AID catalyzes CSR to some extent (Figure 2).

Thyroid-stimulating TRAb is an IgG class antibody [32,37]. Kumata et al. measured TRAb-IgG and TRAb-IgM levels in the sera of 34 patients with Graves’ disease and 15 healthy controls, and noted that serum TRAb-IgM levels were significantly higher than TRAb-IgG levels, in contrast to serum total IgG levels, which were markedly higher than those of other Ig classes [65]. Furthermore, TRAb-IgM levels were significantly higher in the group with higher EA and VCA levels than in the others group, which indicated the secretion of TRAb-IgM under EBV reactivation conditions.

In addition to this clinical study, we cultured PBMCs from 10 Graves’ disease patients and 14 healthy controls and induced EBV reactivation. We measured total IgG and total IgM levels in culture media and confirmed that IgM levels were significantly higher than those of IgG [58]. These findings suggested EBV reactivation-induced Ig production as the source of IgM.

#### 6.4.2. Ubiquitous Ig Production

In the EBV reactivation-induced Ig production system, B cells may produce antibodies in both the circulation and local tissues. Each process in this system may occur following infection by EBV and its reactivation. The system does not require lymphoid tissue, germinal centers, T-cell assistance, or even antigens, including autoantigens. Therefore, antibody production through this system may be ubiquitous.

In the region of lymphocyte infiltration, once EBV persisting in a certain cell begins to reactivate, the infectious virions produced may spread to and infect the surrounding cells. Consequently, the number of EBV-infected cells and regional concentration of antibodies may increase in peripheral tissues.

We found that EBV(+) lymphocytes and IgG4(+) plasma cells accumulated in the same area in 7 out of 11 resected thyroid tissue samples from Graves’ disease patients [66]. These findings indicated that IgG4 was produced under the influence of EBV-reactivation in local thyroid tissue. 

#### 6.4.3. Rescue of Autoreactive B Cells

Circulating B cells enter the lymph nodes through high endothelial venules (HEV) and encounter their specific antigens through afferent lymphatic vessels [63]. B cells digest antigens, present them to cognate CD4+ T cells, are activated, and then proliferate due to CD40 signals from T cells (Figure 3, left).

Autoreactive B cells, which have specificities for autoantigens, have difficulties locating their specific autoantigens because these antigens are packaged inside cells, including the nucleus, DNA and intracellular components [63]. Therefore, autoreactive B cells cannot be activated and are purged from lymphoid tissue (Figure 3, right). They are finally removed and the production of autoantibodies is avoided, which may be one of the peripheral selection processes.

However, when EBV infects autoreactive B cells that need to be removed, B cells may be activated polyclonally, differentiate to plasma cells, and then produce autoantibodies along with EBV reactivation (Figure 3, right). EBV reactivation-induced Ig production is a system that may rescue autoreactive B cells, and the antibodies produced may be skewed toward autoreactivity, which explains not only the increased serum levels of various autoantibodies in EBV primary infection and reactivation, including the acute phase of IM [14,15,16,17], but also the overlap of autoimmune diseases, for example, Graves’ disease and insulin-dependent diabetes mellitus.

### 6.5. Role of Antibodies Induced by EBV Reactivation

The most prominent difference between the antibody-producing system through germinal centers and bone marrow and that induced by EBV reactivation is the presence or absence of germinal centers (Figure 3). The EBV reactivation-induced system does not have germinal centers in its pathway. Although EBV induces AID in host B cells, the efficiency of CSR is moderate, and thus, affinity maturation may also be insufficient. We speculated that the antibodies produced by the EBV reactivation-induced system may have weak affinity for antigens.

**Figure 4:**
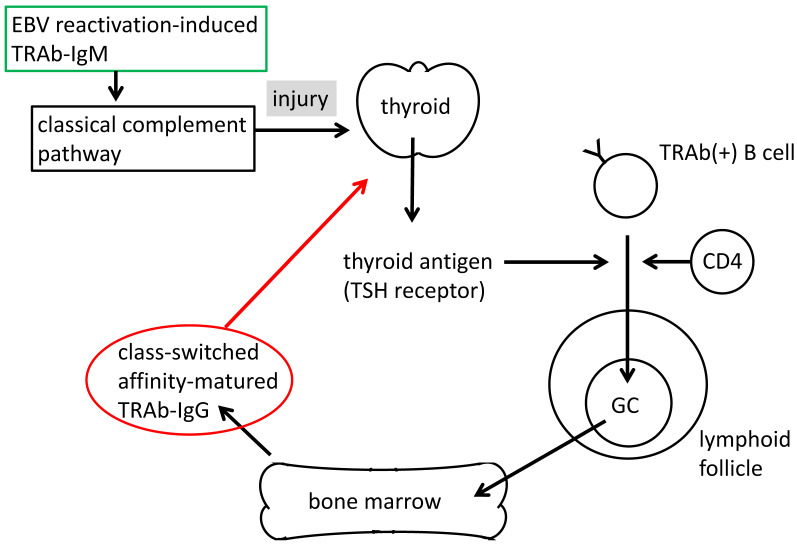
Tissue injury by the TRAb-IgM-activated complement system and production of high affinity TRAb-IgG. EBV reactivation-induced TRAb-IgM may injure thyroid follicular cells through the activation of the classical complement pathway. Thyroid debris may be removed to the circulation and incorporated by B cells in lymphoid tissues. B cells digest and present an antigen of the TSH receptor to cognate T cells, activating the CD40 signal, and proliferated B cells then form germinal centers to produce affinity-matured TRAb-IgG, which may develop and exacerbate Graves’ disease (red arrow).

However, we noted an important role for EBV reactivation-induced IgM-autoantibodies that differed from that of typical autoantibodies (Figure 4). IgM antibodies activate the classical complement pathway and injure target cells. EBV reactivation-induced IgM autoantibodies may injure self-tissue through complement. Small sections of injured tissue may be removed in the circulation and incorporated by antigen presenting cells (APCs), particularly B cells in lymphoid tissues. B cells digest and present tissue antigens to cognate T cells, which activate the CD40 signal, and proliferated B cells then form germinal centers to produce affinity-matured IgG antibodies. Therefore, this tissue injury may lead to immune responses and induce the development and exacerbation of autoimmune diseases, including Graves’ disease.

The EBV reactivation-induced system may also be a source of low-affinity IgG4.

Besides the tissue injury, EBV-reactivation-induced TRAb-IgM may function as a TSHR-blocking antibody. It was reported that stimulating TRAbs were restricted to an IgG1 subclass [37], whereas IgM-containing fractions did not increase cAMP. Another report suggested that TSHR-blocking antibodies were polyclonal [38].

We confirmed that TRAb-IgM produced by EBV reactivation could bind to recombinant human TSHR (manuscript in preparation). In order to clarify the cell injury and TSHR-blocking effect of the antibodies produced by EBV reactivation, we recently separated the antibodies produced by EBV reactivation in culture.

## 7. How the EBV Reactivation-Induced Ig Production System Can Be Connected to the Development and Exacerbation of Autoimmune Diseases

Based on the above results, we consider the relationship between EBV reactivation as follows.

Circulating autoreactive B cells evaded from central selection and partially from peripheral selection must be deleted without producing antibodies. However, once EBV has infected the B cells, they can be activated polyclonally and differentiate to be plasma cells along with EBV reactivation. The rescued B cells are therefore skewed toward autoreactivity (Figure 3), and these are the reason why various autoantibodies are increased in IM and may be the reason for the overlap of autoimmune diseases.

The antibody production induced by EBV reactivation occurs in the circulation and local tissues instead of bone marrow. 

Since these antibodies are in IgM class, they can activate the classical complement pathway and the target cells are injured and removed in the circulation, which lead to the immune response to produce affinity-maturated IgG (Figure 4).

Our study targeted Graves’ disease as an example of autoimmune disease and we could sample such a limited number of the subjects. Therefore, the more detailed study is required targeting many other autoimmune diseases with a number of subjects.

## 8. T Cell Involvement in Autoimmunity

B cells are involved in autoimmunity through the production of autoantibodies, whereas T cells participate with their effector functions.

The specific antigens and contribution of cognate CD4 T cells are required for the activation and proliferation of B cells and subsequent formation of germinal centers. When a CD4 T cell recognizes a specific peptide-HLA complex on a B cell surface, the T cell expresses the CD40 ligand (CD40L) and interacts with CD40 on the B cell. The B cell is then activated, enters a mitotic cycle, and forms a germinal center. B cells undergo CSR and affinity maturation by SHM, in which follicular helper T (Tfh) cells are needed to help B cells [67]. When a naive CD8 T cell receives a T-cell receptor stimulation from a specific peptide-HLA complex, it is activated and becomes a cytotoxic T lymphocyte (CTL) in various manners. CTLs exhibit cytotoxicity and induce apoptosis of their target cells, including EBV-infected cells. Therefore, autoreactive CTLs injure healthy cells and induce apoptosis.

Approximately 10% of the CD4 T-cell population exert inhibitory effects on T-cell activation and are called regulatory T (Treg) cells [68,69]. Treg cells constitutively express CD25, also known as the IL-2R α chain, and form high-affinity IL-2R. Treg cells also strongly express cytotoxic T-lymphocyte antigen (CTLA)-4 through a transcription factor Foxp3 [70]. Treg cells suppress the surrounding T cells by CTLA-4, which competitively binds to co-stimulatory molecules on APC, or by high-affinity IL-2R, which deprives IL-2. Treg cells are reported to be autoreactive [69] and may inhibit the activation of autoreactive T cells, which contributes to the suppression of autoimmunity.

Prior to T-cell reactions, antigen presentation is needed. APCs uptake and process antigens for presentation by HLA molecules. However, before uptake, the tissue must be injured and removed in the circulation. IgM antibodies may be attributed to injured target cells through the complement system (Figure 4).

## 9. Conclusions

We herein proposed an EBV reactivation-induced Ig production system that is an alternative system of antibody production with the following characteristics: IgM dominance, ubiquitous Ig production, and the rescue of autoreactive B cells.

IgM dominance means that EBV reactivation-induced antibodies injure target cells by activating the classical complement pathway. Ubiquitous Ig production does not require germinal centers or bone marrow. The rescue of autoreactive B cells that were to be removed results in a skewed population for autoantibodies, which may be the reason why various autoantibodies appear in IM, and the reason for the overlap of autoimmune diseases.

Antibodies induced by EBV reactivation may play important roles in the development and exacerbation of autoimmune diseases. The study on EBV reactivation-induced Ig production has just begun. Further mechanisms are to be elucidated in the future.

## 10. Patents

Patent Number 6667806: 28 February 2020 (Japan Patent Office).

## Figures and Tables

**Table 1:** Examples of EBV latent and lytic proteins.

**Latent Cycle**		
EBNA1		
EBNA2		
EBNA3A		
EBNA3B		
EBNA3C		
EBNA-LP		
LMP1		
LMP2A		
LMP2B		
**Lytic Cycle**		
BZLF1		Immediate-early
BRLF1		replication
BMRF1	EA	
BALF5		Early replication
BGLF4		
BHRF1		
BCRF1		
BNRF1		Late replication
BFRF3	VCAp18	
BLRF2	VCAp23	
BDLF3	gp150	
BLLF1	gp350/220	

## Abbreviations

EBV	Epstein-Barr Virus
TRAb	Thyrotropin Receptor Antibody
TSH	Thyroid Stimulating Hormone (Thyrotropin)
LMP1	Latent Membrane Protein 1
NF-κB	Nuclear Factor κB
AID	Activation-Induced Cytidine Deaminase
CSR	Class-Switch Recombination
SHM	Somatic Hypermutation
GC	Germinal Center
